# P-2223. Plasma Microbial Cell-free DNA Next-generation Sequencing can be a Useful Diagnostic Tool in Patients with Osteoarticular Infections

**DOI:** 10.1093/ofid/ofae631.2377

**Published:** 2025-01-29

**Authors:** Francesco Petri, Omar Mahmoud, Nischal Ranganath, Said El Zein, Rita Igwilo-Alaneme, Omar M Abu Saleh, Elie Berbari, Madiha Fida

**Affiliations:** Division of Public Health, Infectious Diseases and Occupational Medicine, Department of Medicine, Mayo Clinic College of Medicine and Science, Mayo Clinic, Rochester, MN, USA, Milan, Lombardia, Italy; Division of Public Health, Infectious Diseases and Occupational Medicine, Department of Medicine, Mayo Clinic College of Medicine and Science, Mayo Clinic, Rochester, MN, USA, Milan, Lombardia, Italy; Mayo Clinic, Rochester, Minnesota; Mayo Clinic, Rochester, Minnesota; Mayo Clinic, Rochester, Minnesota, Rochester, Minnesota; Mayo Clinic Rochester, Rochester, Minnesota; Mayo Clinic, Rochester, Minnesota; Mayo Clinic, Rochester, Minnesota

## Abstract

**Background:**

The introduction of shotgun metagenomic sequencing (sMGS) methods that identify the presence of microbial cell-free DNA (mcfDNA) in peripheral blood have shown promising results in various populations of patients. There is a pressing need to evaluate its application for osteoarticular infections (OAIs) in real-world setting.

**Figure:**
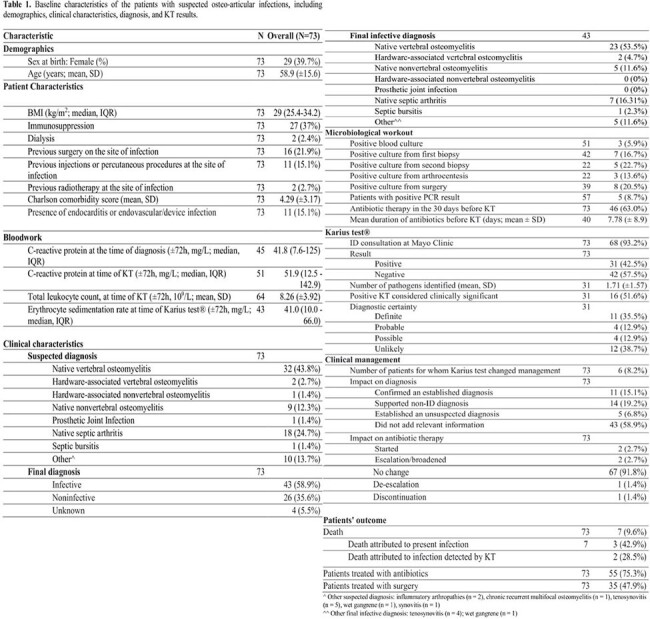

Baseline characteristics of the patients with suspected osteo-articular infections, including demographics, clinical characteristics, diagnosis, and KT results.

**Methods:**

We retrospectively collected data on 73 adult patients who presented to the Mayo Clinic with suspected OAIs between 2019 and 2023, and for whom mcfDNA sMGS (Karius®, KT) testing was part of their diagnostic workup. We collected data on comorbidities, diagnostics, microbiological workout and its concordance with the KT. The primary objective was to evaluate its clinical impact on OAI diagnoses and management distinguishing into four categories: (1) KT was able to confirm an established diagnosis, (2) KT supported non-ID diagnosis, (3) KT established an unsuspected diagnosis, (4) KT did not add relevant information.

**Figure:**
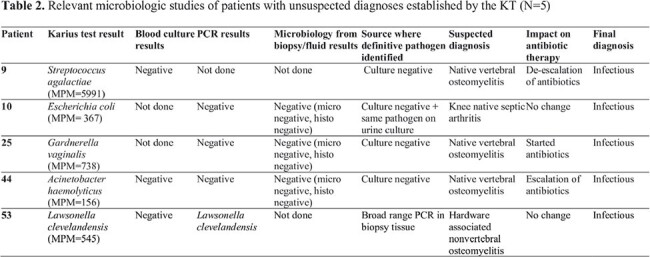

Relevant microbiologic studies of patients with unsuspected diagnoses established by the KT (N=5)

**Results:**

In our cohort KT was performed in 73 patients (Table 1). Among the infected individuals, KT yielded positive results in 22/43 (51.2%) cases. Out of these 22 cases, 11 (50%) showed agreement with conventional diagnostic workup, while in five (22.7%) cases, the KT established an unsuspected diagnosis, improving the diagnostic power from 11/43 (25.6%) to 16/43 (37.2%) (Table 2). Moreover, yielded a negative result in 19/26 (73.1%) patients with non-infectious diagnosis (Figure 1). Native Vertebral Osteomyelitis (NVO) diagnosis (p < 0.001) or OAIs with concomitant presence of endocarditis or endovascular infection (p = 0.005) were statistically associated with a definite, probable, or possible diagnostic certainty of KT result (Table 3). Overall, KT influenced antibiotic therapy in six cases (8.2%).

Table 3

**Figure:**
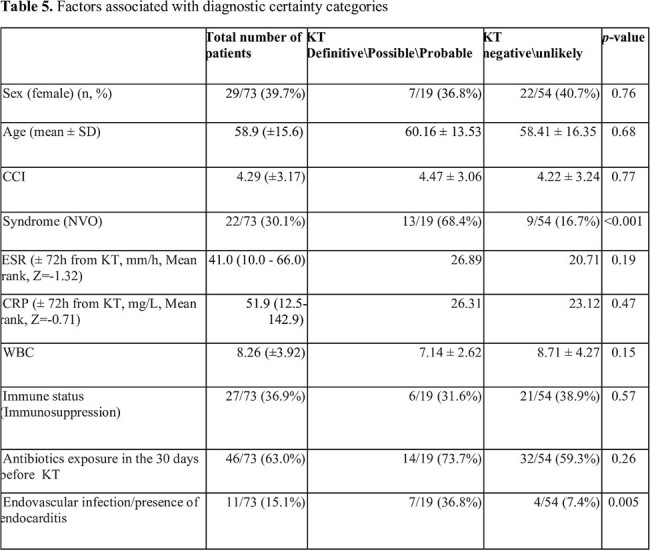

Factors associated with diagnostic certainty categories

**Conclusion:**

This is the first retrospective study aiming to assess the real-world usefulness of the KT on the clinical management of patients with OAIs, with a special focus on NVO. It underscores KT's real-world application in cases where traditional diagnostic methods fall short. In highly complex OAIs cases, KT added 11.6% to the diagnostic power by establishing unsuspected diagnosis in 5 of 43 infective patients. Patients with NVO or OAIs with concomitant endocarditis or endovascular infections particularly benefited from KT testing.

Figure 1

**Figure:**
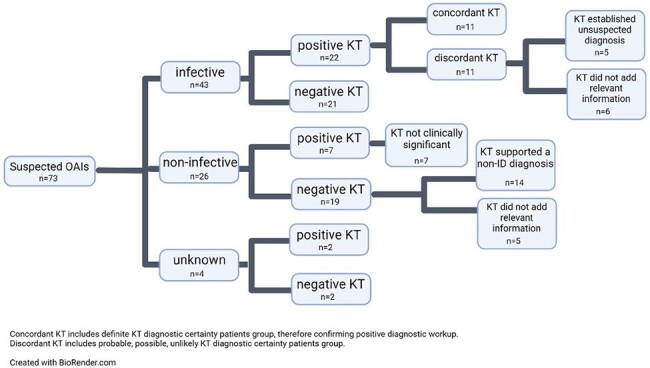

Distribution of KT results and their respective outcome according to the final diagnosis of suspected OAIs cases.

**Disclosures:**

All Authors: No reported disclosures

